# Autism Spectrum Disorder in Females and Borderline Personality Disorder: The Diagnostic Challenge

**DOI:** 10.7759/cureus.40279

**Published:** 2023-06-12

**Authors:** Sofia Pires, Pedro Felgueiras, Sandra Borges, Joana Jorge

**Affiliations:** 1 Child and Adolescent Psychiatry, Centro Hospitalar de Vila Nova de Gaia/Espinho, Vila Nova de Gaia, PRT; 2 Psychiatry, Centro Hospitalar de Vila Nova de Gaia/Espinho, Vila Nova de Gaia, PRT

**Keywords:** diagnostic, personality, borderline, female, autism

## Abstract

Autism spectrum disorder (ASD) is a heterogeneous neurodevelopmental disorder characterized by pervasive deficits in communication and social interaction and patterns of repetitive, restrictive interests and/or stereotyped behaviors. Female sex/gender is not represented in the current conceptualization of ASD, and there is emerging evidence of a female phenotype. The etiology of ASD and borderline personality disorder (BPD) is not fully understood. Clinical observations suggest that ASD and BPD can overlap in clinical presentation and diagnostic characteristics, especially in female ASD cases.

We report two clinical cases of two adolescent girls presenting overlap symptoms between ASD and BPD, raising questions about the female ASD phenotype and the potential misdiagnosis of ASD characteristics with BPD, as well as its impact on diagnosis and management.

Diagnostic differentiation is crucial for targeted therapeutic interventions (psychopharmacological and psychosocial). Further studies are needed to enlighten the clinical similarities and diagnostic overlap between ASD females and BPD.

## Introduction

Autism spectrum disorder (ASD) is an heterogeneous neurodevelopmental disorder characterized by pervasive deficits in communication and social interaction and patterns of repetitive, restrictive interests and/or stereotyped behaviors [1]. Behind these core criteria, there is considerable heterogeneity in clinical presentation [2].

The prevalence of ASD is estimated to be one in 36 and a documented male-to-female ratio of approximately 3/4:1 [3,4]. However, recent studies have found that it is likely to be lower [3,4]. Recent studies have found that this ratio is not consistent, suggesting that diagnostic sex/gender bias (both terms - sex and gender - together are used when referring to differences between boys and girls to inform that it is not clear which is the role of biological sex or social gender or both) exists and that ASD girls are being underdiagnosed, misdiagnosed, or diagnosed later in life [5]. Female sex/gender is not represented in the current conceptualization of the ASD, and there is emerging evidence of a female phenotype with unique symptomatology, different clinical presentation, and associated comorbidities [2,6,7]. Most diagnostic assessments and tools are based on research with autistic males and underestimate symptoms or are not sensitive enough to autistic female presentation, contributing to sex/gender bias [7,8].

Borderline personality disorder (BPD) is characterized by a pervasive pattern of instability of interpersonal relationships, self-image, and affective instability, including emotional dysregulation, malfunction in interpersonal relationships, and difficulty in impulse control. It is documented as having similar gender prevalence in the general population; however, in clinical samples, BPD is more commonly diagnosed in females [1,9].

The etiology of ASD and BPD, including the contribution of genetic and environmental factors, is not fully understood [9]. Clinical observations suggest that ASD and BPD can overlap in clinical symptoms and diagnostic characteristics, especially in female ASD cases with preserved cognitive functioning, leading to differential diagnosis uncertainty [9,10].

This article aims to explore the female ASD phenotype and the potential misdiagnosis of ASD characteristics with BPD, as well as its impact in diagnosis and management.

We report two clinical cases that raised doubts between ASD/BPD diagnosis and a review of the state of the art with database research.

## Case presentation

The following cases were considered to explore the topic.

Clinical case one 

Fourteen-Year-Old Female Adolescent Described as Emotionally Dysregulated, with Unstable and Rigid Personality Traits

She had a history of non-suicidal self-harm with arm cuts and food restriction (multiple emergency room episodes). In regards to neurodevelopmental data, when in preschool, she presented a peculiar sensory processing profile with difficulties in tolerating some textures and increased sensitivity to noise. She was described as shy and frequently hid herself to play alone without any other alarming signs during the development period.

She was in the eighth year of school, regular, with good grades. She identified three “not-so-close” friends but she didn’t like school and preferred to avoid social contact. She lives with her mother and sister. Her parents separated ten years before the first appointment.

During the appointments, she presented with oscillating mood between sadness, irritability, emotional lability, “emptiness” and “loneliness” feelings, food restriction, binge eating, non-suicidal self-harm episodes and frequent worries about peer relationships, self-image, and the “need to be better and do things better” to fit in. 

She had a bizarre rigid posture, with disharmonic body movements, superficial affect and oscillating mood, not resonant, and a very low tone of speech. She was initiated on antidepressant and psychotherapeutic intervention. She did not respond to treatment and maintained her difficulties. 

The psychological evaluation concluded that, besides her peculiar posture and developmental history, she had a rigid social functioning resulting in dysregulation and isolation, with a lack of spontaneity in affection demonstration, difficulty in symbolic interpretation, and central coherence/theory of mind difficulties. She also showed depressive feelings related to her difficulties in social interaction and communication, with feelings of insecurity, inferiority, and a constant need to correspond to society expectations. 

Clinical case two

Fifteen-Year-Old Female at a First Contact with Child and Adolescent Psychiatry (CAP) Appointment 

She lives with her parents and younger sister, and she is attending the tenth year of school with excellent grades. 

Since 14 years old, she manifested an unstable mood and behavior profile with irritability and aggressive episodes, impulsivity, and highly emotional reactivity associated with adaptation difficulties. Primary care psychological observation registered it as “less healthier personality traits'' before referral to CAP.

She was described as an introverted child with no symbolic play. She showed little interest in other children and some particular and repetitive interests like reading and history (“she spent hours watching the same TV episode”).

She presented with peculiar contact; excessive expressiveness; sometimes theatrical, frequent emotional lability; and affective instability with low resonance. She was always focused on her relationship difficulties with peers, with concrete language and particular prosody. In later appointments, she was found to have more negative and depressive feelings related to her social failure.

The psychological evaluation concluded that, in addition to her developmental clues and unusual body posture often oscillating with stiffness, she felt like an outsider, uncomfortable, and different from her peers. She also presented a concrete thought profile, routine rigidness, and difficulties dealing with emotions.

## Discussion

To better understand the female ASD phenotype, many different factors need to be taken into account, when considering the diagnosis.

 Autistic girls tend to collect information on people, rather than objects, and they present restrictive/repetitive interests that are considered “gender-normal” (e.g., dolls, jewelry, craft, books, art, and animals) [4]. Some studies also reported differences in what concerns these behaviors and/or interests, stating that they are less common in ASD girls versus ASD boys and that they can be less recognized [5,11]. In terms of behavior, parents and teachers tend to interpret passivity, calmness, and shyness as female gender-appropriate [12]. On the other hand, boys present more frequently with externalization behaviors such as aggressiveness, hyperactivity, and inattention [4,12]. In both clinical cases, girls were described as “shy” and “introverted,” being gender-normalized and even complimented by parents and teachers, and their interests (even if with a repetitive/restrictive pattern) were not taken as unusual. 

ASD girls are also more interested in social relationships and have more social awareness, vocabulary words related to emotions and desire for interaction, compared with ASD boys [4]. With regard to communication, they have pretend play and imagination, but in a repetitive and controlled way and with no reciprocity; they have higher linguistic abilities but have difficulty to socially adequate them [8,13].

The female figure is traditionally associated with emotivity, communication, nurture, sensitivity, and compassion. In opposition, the male image is represented by logic, strength, physical capacities, and rationality [12]. Girls with autism tend to feel pressured to engage in these behavior patterns and social-constructed ideals when they are not accepted among their peers [12]. In order to simulate typical girls and evade bullying and social discrimination, ASD girls mask or *camouflage*, pretending not to show autism features [14].

*Camouflage* is a unique phenomenon, particularly relevant in high-functioning autistic females skilled at observing, imitating, and mimicking their peer behaviors [7,14]. It refers to behaviors or coping strategies used to hide ASD characteristics and try to fit in [4]. Some examples of *camouflage* may include active and discomforting eye contact (attempt to make and maintain eye contact), excessively using jokes and learned phrases, changing speech volume or intonation, and imitating nonverbal language inadequately [4]. In both clinical cases, we can observe these behaviors: bizarre postures (stiffness and swinging), disharmonic body movements, superficial affects, peculiar contact and tone of speech, and excessive expressiveness, sometimes theatrical. 

As they usually describe in their words, “pretend to be normal” is not natural and requires cognitive effort and focus, leading to exhaustion [4]. They end up losing their identities, interfering in relationships and mental health, with a strong feeling of “non-belonging” [12]. The intense desire to please others may make them more vulnerable to abuse, exploitation, victimization, and social naiveté [4,12]. These non-natural and forced behaviors (*camouflage*) driven by social anxiety differ from social skills training, which aims to develop successful and natural social interaction throughout a positive internal and emotional experience [4].

ASD girls tend to have a higher rate of comorbid conditions such as anxiety and depression [4,7]. They may be exposed to a double-hit risk: typically developing females have approximately twice the risk of depression at adolescence and ASD peers may be at exceptionally high risk of internalizing disorders (anxiety, depression, eating disorders) [4]. As shown in clinical case two, the girl was so focused on her relationship difficulties with her peers that in late appointments she ended up presenting with more negative and depressive feelings related to her social failure. 

Female ASD and BPD, as exposed above, may have a basis of phenomenological resemblances (not common etiology or pathogenesis) [15].

Impairments in the theory of mind (the ability to represent others' mental states and how we respond to them) and lack of empathy seem to be the basis of social dysfunctionality in ASD [10]. Empathy is defined as an integration of two dimensions: affective empathy (the experience of feelings and emotions of others) and cognitive empathy (the understanding and ability to adopt another person’s perspective and to judge and understand intentions in order to control its own) [16]. This last construct is consistent with the theory of mind’s definition and seems to be impaired in BPD individuals [10]. Heightened affective empathy without cognitive coherent mental integration of other’s mental and emotional states results in relationship dysfunctionality or emotional disturbance - "the borderline empathy paradox” [17]. According to Alan Krohn, this paradoxical impairment is reactive to a confused or negligent parenthood with consequent hypervigilance, in order to survive in an unstable and dangerous environment [17]. There is evidence that on this basis, ASD and BPD have similar difficulty in emotional interpretation and recognition [15]. Recent studies also suggest comorbidity between ASD and BPD and the significative presence of autistic traits (self-reported) in BPD individuals [10,15].

Both ASD and BPD individuals can apparently present a similar pattern of interpersonal relationships, identity disturbance, impulsivity, affective lability, and difficulty controlling anger [15]. The *acting out *instead of verbalizing emotions, intense and dysfunctional relationships and superficial friendships in BPD, and social/quality communication difficulties, the effort of adapting to social norms and expectations in female ASD can phenotypically overlap and turn diagnostic evaluation into a challenge [10,15]. In both clinical cases we can read the description of these behaviors, and in clinical case two, the primary care psychologist even suggested “less healthier personality traits'' (which is very vague and unspecific but he was identifying a strange behavior pattern). 

It is important to correctly diagnose in order to address proper interventions and management (social-centered training specific for ASD) and to overcome difficulties considering basic psychopathology (as an example: self-harm in ASD tends to be associated with sensory overload while BPD tends to relate with affective instability and emotional dysregulation) [9]. Moreover, the comorbidity between ASD and BPD might also be associated with higher suicide risk [9].

Despite being very similar, there are still many particularities differentiating ASD females and BPD. Clinical and developmental history as well as clinical characteristics, symptoms, and a thorough psychological evaluation must always be taken into account.

## Conclusions

Obtaining a diagnosis promotes a sense of belonging within a community and an improved self-view. ASD remains a diagnostic challenge, particularly when considering sex/gender differences and its potential overlap with BPD. Diagnostic differentiation is crucial toward targeted therapeutic interventions (psychopharmacological and psychosocial).

BPD and female ASD can phenotypically overlap, and the clinician must be alert in order to manage a complete and thorough evaluation. It is still difficult to draw accurate conclusions based on the recent literature. Research in sex/gender difference is still limited, with heterogeneous results, and further studies are needed to enlighten the clinical similarities and diagnostic overlap between ASD females and BPD. Future research should use reliable, accurate measures focusing on sex differences in autism and females with ASD. It should also be focused on adapting diagnostic instruments (which do not have the construct including the female autism phenotype).
